# Total Knee Arthroplasty in Octogenarians: Should We Still Be so Restrictive?

**DOI:** 10.3390/geriatrics6030067

**Published:** 2021-06-30

**Authors:** Jose Maria Trigueros-Larrea, Maria Antonia Gonzalez-Bedia, Jose Maria Lomo-Garrote, Oscar Martin-de la Cal, Miguel Angel Martin-Ferrero

**Affiliations:** 1 Orthopaedic Surgery and Traumatology Department, Hospital Clinico Universitario Valladolid, 47005 Valladolid, Spain; magonzalezbed@saludcastillayleon.es (M.A.G.-B.); miguelangel.martin.ferrero@uva.es (M.A.M.-F.); 2 Centro ORIGEN, Diagnostico y Traumatologia, Department of Traumatology, 47006 Valladolid, Spain; j.lomo@realvalladolid.es; 3 Scholar FUNGE Reseach Unit, University of Valladolid, 47002 Valladolid, Spain; Oscar.martin@sanviatorvalladolid.com

**Keywords:** older person, total knee arthroplasty, outcomes, comorbidities, octogenarians

## Abstract

Demand for total knee arthroplasty (TKA) in octogenarians will increase in subsequent years as society ages. We conducted a retrospective observational study in octogenarians operated on with TKA between 2015 and 2019, comparing preoperative and postoperative Knee Society Score (KSS), Knee Society Function Score (KSFS), extension and flexion balance, and radiologic alignment using a paired Student t-test. A chi-squared test was used to correlate mortality with Charlson comorbidities index score and with ASA scale. Kaplan–Meier analysis was performed to calculate patient survival. In this period 36 patients ≥80 years underwent TKA, with a mean age of 81.6 years. Of these, 24 patients (66.7%) were classified as ASA II and 12 (33.3%) as ASA III. Sixteen patients (44.4%) were Charlson 0, 14 (38.9%) Charlson 1, two (5.6%) Charlson 2, and four (11.1%) Charlson 3. KSS, KSFS, flexion and extension range, and radiologic alignment were statistically significant (*p* < 0.001) when comparing preoperatory and post-operatory data. No correlation (*p* > 0.05) was found between mortality and ASA or Charlson score. Seven patients (19.4%) suffered a medical complication and two patients experienced surgical complications. Four patient died (11.1%) during follow-up. The mean patient survival was 67.4 months. Patients ≥80 years achieve clinical improvement after TKA. Comorbidities, not age, are the burden for surgery in older patients.

## 1. Introduction

Our society is aged. In Spain, life expectancy is 83.19 years and 2,856,101. persons are over 80, which represents 6% of the population, and this will increase in the coming years [1]. Older people, today, are more active and demanding about improving their quality of life. Orthopedic surgeons should provide the best care for these patients to reach their expectations. Total knee arthroplasty (TKA) is a successful and cost-effective treatment for patients with end-stage knee osteoarthritis, when conservative measures have failed. [2]. TKA provides pain relief and improves functional status and quality of life, with low perioperative complications [3]. In the United States, the projection of the increase in the volume of TKA by 2030 is a growth of 85% from 2014 [4], mainly due to the aging the population. 

A recent systematic review [5] showed better patient related outcome measurements in the early 70s and no differences in revision and mortality rate between 70 and 80. There is a general notion among surgeons that patients over 80 are bad candidates for knee replacement, with bad outcomes, high morbimortality risk, and complications, and that patients over 80 usually reject to surgery. Although some studies have reported increased mortality and complication rates in older patients [6,7], many studies have found the same results in function, radiographic outcomes, pain relief, satisfaction, and improvement of quality of life as in younger patients [8,9,10], even in nonagenarians [11,12,13]. Moreover, knee replacement surgery has evolved and is now safer. The last decade has seen a revolution in perioperative patient management: preoperative medical optimization allows patients to go to surgery in better condition, systematic use of tranexamic acid has decreased dramatically the transfusion rate, local infiltration anesthesia and selective sensitive nerve blocks allow the early walking of patients. All these changes are part of the enhanced recovery programs designed for optimizing the whole process of TKA, initially for healthy patients, but some studies have reported that older patients have the most to gain from these programs [14,15].

Kuperman et al. [16] in his meta-analysis concluded that data supports offering primary total knee arthroplasty to select geriatric patients, although the risks of complications may be increased. Andreozzi et al. [17] reported that comorbidities rather than age affect outcomes in octogenarian patients after TKA.

Our main aim was to evaluate the outcomes in patients ≥80 years after a TKA operation at our institution. Our secondary objective was to identify the prevalence of adverse events during hospitalization and the survival rate.

## 2. Materials and Methods

We conducted a retrospective observational study of patients over 80 years, operated on with knee arthroplasty at the Clinic University Hospital of Valladolid by two exclusive, dedicated knee surgeons (J.M.T-L. and J.M.L-G) from 1 January 2015 to 1 March 2019. Exclusion criteria were fracture, sequelae of septic arthritis, revision surgery, or less than 1 year of follow up. This study was designed respecting the STROBE guidelines for observational studies.

Deceased patients were included in the analysis, as mortality was one of the variables for the study. 

Age, sex, side, comorbidities, anesthesia scale of the American Society of Anesthesiology (ASA), length of stay (LOS), surgical data, and complications were obtained from the medical file records, and Charlson comorbidities index score [18] was calculated retrospectively. The Charlson index includes 19 comorbidities, and scores with a single number patient’s risk for surgery. We reported preoperative and postoperative status using the Knee Society Score (KSS) [19], evaluated pain, range of motion, flexion contracture, extension lag, antero-posterior and medio-lateral stability, alignment, and Knee Society Function Score (KSFS), which specifically evaluates walking, stairs, and need of aids to walk. Patients visited in office preoperatively and 1 month, 3 months, 6 months, and 1 year after surgery, and then annually afterwards. Radiologic data, expressed as hip-knee-ankle angle, were obtained from a weightbearing full leg x ray, and diaphyseal femorotibial angles were measured as needed for the KSS.

All patients were operated on by the two aforementioned surgeons under spinal anesthesia; antibiotic prophylaxis with 2 g IV cefazolin preoperatively and repeating 3 doses of 1 g IV cefazolin every 8 h during the first 24 h, except in case of allergy to betalactamics; administering 400 mg IV teicoplanin and another dose 12 h later. Medial parapatellar approach with isquaemia with tourniquet was used in all cases. We implanted a cemented Triathlon posterostabilized Knee arthroplasty (Stryker, Kalamazoo, MI, US) using the measured resection technique or cemented VEGA posterior stabilized knee arthroplasty assisted by Orthopilot navigation system (Aesculap; Bbraun, Tuttlingen, Germany) using a gap balancing technique at the surgeon’s discretion. An epidural catheter was used for pain control until we introduced a local infiltration anesthesia (LIA) protocol in 2018. We started to systematically use tranexamic acid 10 mg/kg before releasing the tourniquet 3 h later in 2017. We obtained from the patients an informed consent to participate in the study, and approval from the local Hospital Clinical Research Ethics Committee was obtained, with the number PI 21-2336

Statistical analysis was performed using the institutional Statistical Package for the Social Science (SPSS) software version 24.0 for Windows (IBM, Armonk, NY, USA). Continuous variable were expressed as mean ± standard deviation (SD). Categorical variables were displayed as absolute frequencies and relative frequencies as percentages. Radiological alignment, range of motion, KSS, and KSFS were compared pre- and post-operatively using a paired Student t-test for parametric data and chi-squared test for finding any correlation in mortality and Charlson score, and between mortality and ASA scale. A *p* value of less than 0.05 was considered statistically significant. A Kaplan–Meier analysis was performed to calculate patient survivorship, with 1 March 2021 as a cut-off date. The end point criteria were aseptic implant loosening or death, excluding infection or revision for fracture from failures. 

## 3. Results

### 3.1. Patients characteristics

A total 389 TKAs were performed between 2015 and 2019 by the aforementioned surgeons in our institution. After excluding patients under 80 years, 38 patients were available for the study. One patient was excluded because the indication for knee replacement was a fracture of the lateral condile in a severely degenerative knee. Another patient was lost to follow up at the 3-months postoperative scheduled visit. Finally, 36 patients, 21 women and 15 men, where included for analysis. Patient characteristics and comorbidities are listed in Table 1. Mean follow up was 50 months (min 14, max 73). Right side was involved in 17 patients and left side in 19. The mean age of the patients who underwent TKA at the time of surgery was 81.6 years (SD ±1.5), ranging from 80 to 86 years. 

### 3.2. Comorbidities 

Comorbidities are listed in Table 1. 

### 3.3. Clinical Outcomes

The KSS, KSFS, flexion range, extension range, and radiologic alignment were statistically significant when comparing preoperatory and post-operatory data, and are shown in Table 2. The correlation between mortality and ASA score or Charlson score did not reach statistical significance (*p* > 0.05). Twelve patients (33%) needed walking aids (one patient used 2 canes, eleven 1 cane) preoperatively, while only five (13%) needed postoperative aids, where two patients used one cane due to contralateral pain, and another three used a cane to prevent falls. 

### 3.4. Complications

Complications are summarized in Table 3.

#### 3.4.1. Medical Complications

During hospitalization seven patients (19.4%) suffered from a medical complication, summarized in Table 3. 

#### 3.4.2. Surgical Complications

One patient had persistent drainage through the wound that required negative pressure therapy, and healed uneventfully. Another patient in the 3 month scheduled visit presented with severe pain and deformity, showing a fracture of the medial condile with femoral component mobilization that required revision surgery with a semi-constrained CCK implant and distal augments, with good outcomes at the last visit, 4 years post-operation. Length of stay was 5.8 days (SD ±1.8 days), ranging from 3 days to the 14 days of the patients that needed negative pressure therapy.

### 3.5. Mortality 

**Figure 1:**
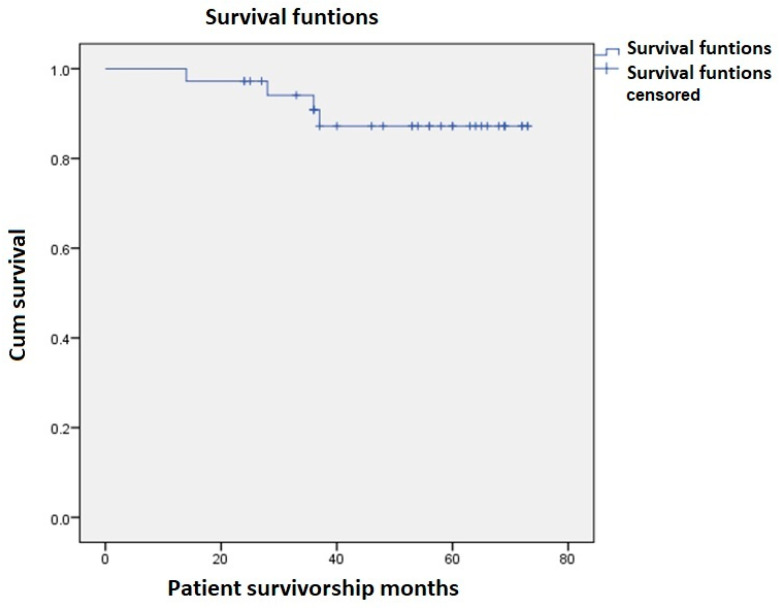
Kaplan–Meier patient survivorship.

Four patients (11.1%) died up to 1 March 2021. Causes of deaths were acute congestive heart failure 3 years after replacement, pneumonia 37 months after surgery, B-cell lymphoma 28 months postoperatively, and a patient diagnosed with a bladder carcinoma 2 years before the operation who died 14 months after operation, due to acute kidney failure due to the urological tumor. The estimated mean patient survivorship was 67.4 months (SD ± 2.627 months). The Kaplan–Meier analysis of patient survivorship is shown in Figure 1.

## 4. Discussion

A patient aged 80 or over, demanding a TKA is an increasing clinical occurrence in our daily practice. Actually, we have a restrictive indication for TKA in healthy patients over 80 years or those operated on for the contralateral knee that assumes their risk. We cope with many patients whose comorbidities, sometimes cardiac diseases, are well established and controlled who demand a TKA, so we decided to conduct this study to improve our state-of-art. There is a low evidence quality for studies for this population group, many of them mixing hip and knee arthroplasty. On the other hand, the concept of “older” has evolved over time, and some studies that reported results in older subjects included patients under 80 years. The main aim of our study was to evaluate our clinical results in these patients. KSS, KSFS, flexion and extension range, and radiological axis reached statistical significance. 

Clement N.D. et al. [7] reported a prospective study of TKA in 185 patients over 80 compared with 492 patients with ages ranging from 65 to 74. They showed a statistically significant increase from the preoperative period in Oxford Knee Score (OKS) and SF-12, with no difference between groups, except for SF12 physical score. There was an increased risk of 1 year mortality of 2.5% vs. 1.4%, but this did not reach statistical significance. 

Lizaur-Utrilla A et al. [8] prospectively compared 143 octogenarian patients with a mean age of 83.1 (80–89 years) and 149 septuagenarians during a mean follow up of 3.2 years (2–5 years). They found a significance increase in KSS and KSFS from preoperative to postoperative but no difference between groups, although with a greater gain in the octogenarian group. They also found increases from the preoperative period in WOMAC, SF-12, and VAS, with no differences between groups, except for SF-12 mental score, which was better in octogenarians. 

Andreozzi V et al. [17] retrospectively reviewed 206 matched-paired patients younger than 75 years and older than 80 years. A significant improvement in postoperative knee scores (KSS, KSFS, OKS) was achieved, although without statistical difference between groups. The most important finding of this study was that comorbidities had a greater impact than age on medical complications, where the Charlson comorbidities index was an independent predictor for postoperative cardiac events, delirium, and transfusion rate. 

Murphy BPS et al. [9] compared 359 patients ≥80 years to 2479 patient under 80. Both groups reached a significant raise in SF-12, with no clinical differences between groups. Klasan A et al. [10] retrospectively reviewed 734 TKAs in 644 patients over 80 years with a matched compared group of 644 patients under 80 with a mean follow up of 93.1 months. Both groups experienced a significant gain from preoperative in OKS, but this was lower in the older group. Even in nonagenarians, good clinical results have been reported [9,10,11] despite mortality, complication rates, LOS, readmission rate, and postoperative adverse events [20,21], so surgical counseling with the patient and family should be especially well balanced. 

Medical complications and mortality increase in older patients [7,8,9,10,11,14,17,20,21]. Kuperman et al. [16] reported in a recent meta-analysis that geriatric patients had a small increase in rates of mortality, myocardial infarction, deep vein thrombosis, and LOS, with no differences in improvement in pain and functional score, compared with younger patients. This increase in mortality was not related to perioperative risk, but is due to the decrease of life expectancy in older people. In our study there was only one patient with worsening of previous chronic congestive heart failure and one with an intraoperative onset atrial fibrillation. Four of thirty-six patients died (11.1%) during follow up, in all cases secondary to common diseases not related to surgery. Conversely to Andreozzi et al. [17], we did not found any correlation between Charslon index and morbimortality, probably due to our small sample size.

Transfusion rate has been correlated to age [22]. In our study, two patients out of 36 (5.5%) needed postoperative blood transfusion. Both were operated on before the systematic use of tranexamic acid from 2017. Tranexamic acid has been shown to decrease the need for blood transfusion [23] and the use of navigation is associated with lower blood loss [24].

The length of stay in our study was 5.8 days, which is very similar to that published in recent years (17). Starks et al. [14] reported a similar LOS with enhanced recovery programs that can be successfully used in older population and that these patients have the most to gain from the multidisciplinary recovery programs. Pitter et al. [15] published a shorter LOS, 4 days, with a fast-track program in patients over 85 years than ours. Bovonratwet et al. [25] studied the safety of TKA in patients over 80 years, with a LOS ≤ 1 day compared to younger patients. They found that octogenarians a had higher 30-day readmission rate, with higher medical complications correlated to ASA ≥ 3, not to age. They concluded that in spite of the fact that octogenarians can be discharged on the same day, they may need more post-discharge facilities to reduce medical complications.

Most studies [6,7,8,9,10,11,12,13,17], as ours, used posterostabilized implants for older people, to give a better ligament balance, as soft tissues are more lax. Although this is very common, for some types of patient, generally active males from rural zones, with good bone and soft tissue quality and unicompartimental osteoarthritis, unicompartimental knee arthroplasty (UKA) could be a very good option. Ode et al. [26] showed that UKA is a less invasive procedure that allows early recovery with a low complications rate compared to TKA in patients over 85 years. Although we do not have any experience with older patients; given a adequate indication: unicompartimental osteoarthritis, deformities under 10°, and intact cruciate ligaments, UKA could be beneficial for older people with a lower complication rate, and it is an interesting option. Gustke et al. [27] reported good outcomes in older patients given all-polyethylene tibial designs, with no revision for aseptic loosening and a 33% cost saving of the implant. This kind of tibial design may be an interesting option, though limited to older and low-demand patients for economic reasons. 

This study has several limitations that should be noted. First, our follow up is short for an arthroplasty study, with a mean 4 years and 2 months and a minimum of 1 year. All the patients except one, who died 14 months after surgery, had a minimum of 2 years of follow up. Taking into account that life expectancy in Spain is 83.19 years we consider that our patients overcame this age as a mean, so the results of this study are representative. Moreover, a longer follow up may have some bias due to changes of practice as surgical treatment and especially perioperative anesthesia evolve. A second limitation is the small sample size and the use of two different implants with different surgical techniques, conventional and navigated TKA by two surgeons; we did not study differences between implants, nor between surgeons, because the samples were too small. Finally, another limitation is a selection bias for very healthy patients for their age, so the conclusions of the study may only be applicable to patients with low comorbidities.

In spite of these limitations, the present study reports valuable data with clinical relevance in the short to medium term for patients over 80 years receiving a TKA, and provides data of the type of patients we are selecting for surgery, while it is a starting point towards changing our restrictive criteria, considering age only as a number, and giving more importance to comorbidities for rejecting surgery.

## 5. Conclusions

Patients 80 years and older achieved a large clinical improvement after TKA. In spite of increases in medical complications, no patient should be rejected for surgery due to their age. Comorbidities, not age, are the burden for surgery in older patients. Preoperative medical optimization can decrease complication rates.

## Figures and Tables

**Table 1:** Patients Demographics and Preoperative Medical Comorbidities.

Characteristics	Count (n = 36)/Mean	Percent/SD
Age at surgery	81.6 years (80–86)	±1.5 years
Follow up (months)	50 months (14–73)	
Sex (Male/Female)	21/15	58.3%/41.7%
Side (Left/Female	19/17	52.8%/47.2%
ASA Scale	II 24 (66.3%) III 12 (33.7%)	II 66.3% III 33.7%
Charlson		
0	16 (44.4%)	44.40%
1	14 (38.9%)	38.90%
2	2 (5.6%)	5.60%
3	4 (11.1%)	11.10%
**Comorbidities**		
Hypertension	17	47.20%
Dyslipidemia	14 (41.6%)	41.60%
Benign Prostatic Hypertrophy	6 (16.6%)	16.60%
Cardiac disease	5 (13.9%)	13.90%
Obstructive Pulmonary disease	4 (11.1%)	11.10%
Cerebrovascular ischemic disease	4 (11.1%)	11.10%
Mental health disorders	3 (8.3%)	8.30%
Malignant tumor	2 (5.3%)	5.30%
Deep venous thrombosis	2 (5.3%)	5.30%
Chronic renal disease	2 (5.3%)	5.30%
Polymyalgia Rheumatica	1	2.70%

**Table 2:** Outcome Measures.

	Preoperative ^*^	Postoperative ^*^	*p* Value
KSS	34.6 ± 9	89.9 ± 6.9	*p* < 0.001
KSS function	33.9 ± 10.2	86 ± 11.97	*p* < 0.001
Flexion	101.5° ± 10.87°	106.2°± 9.1°	*p* < 0.001
Extension	−4.2° ± 4.2°	0.97° ± 2°	*p* < 0.001
Radiologic alignment	6.75° ± 6.45°	1.36° ± 1.4°	*p* < 0.001

* Mean ± Standard Deviation.

**Table 3:** Postoperative Complications.

	Count (n = 36)/Mean
Length of stay in days	5.8± 1.84
**Medical complications**	**7**
Confusion	2
Atrial fibrillation	1
Urinary tract infection	1
Urinary incontinence	1
Hematuria	1
Congestive heart failure	1
Anemia requiring transfusions	2
**Surgical complications**	**2**
Persistent wound drainage	1
Fracture and mobilization	1
of the femoral component	

## Data Availability

The data presented in this study are available on request from the corresponding author.
